# Elucidating the Molecular Mechanisms of *Angelica sinensis* Against Hepatocellular Carcinoma Through Network Pharmacology, in silico Molecular Docking, and in vitro Validation

**DOI:** 10.5812/ijpr-169852

**Published:** 2026-05-20

**Authors:** Huajun Wu, Ping Luo, Chongming Shen

**Affiliations:** 1 Department of Pharmacy, Wuhan Third Hospital Tongren Hospital of Wuhan University, Wuhan Hubei, China; 2 Department of Pharmacy, The First People's Hospital of Jiangxia District, Wuhan City, China; 3 Department of Acupuncture and Moxibustion/Tuina, Ningbo Yinzhou No.2 Hospital, China

**Keywords:** *Angelica sinensis*, Hepatocellular Carcinoma, Network Pharmacology, Molecular Docking, Cytotoxicity, Apoptosis

## Abstract

**Background:**

Hepatocellular carcinoma (HCC) is a lethal liver malignancy associated with substantial morbidity and mortality and remains a major socioeconomic burden worldwide. Given the high risk associated with HCC, novel therapeutic approaches are urgently needed. *Angelica sinensis* is a traditional Chinese herbal remedy that contains bioactive phytochemicals with promising anticancer properties, making it a potential candidate for evaluation in HCC.

**Objectives:**

This study integrated computational network pharmacology, molecular docking, and in vitro cellular assays to elucidate the mechanisms by which *A. sinensis* exerts anti-HCC effects in Hep-G2 cells.

**Methods:**

Phytochemicals were identified using the Traditional Chinese Medicine Systems Pharmacology database and filtered according to drug-likeness and oral bioavailability criteria. Key compounds (β-sitosterol, alpha-cephalin, sitogluside, and stigmasterol) were further analyzed using the SuperPred Target Prediction tool to predict potential targets. HCC-related targets were curated from GeneCards and refined using the GeneCards Inferred Functionality Score. The overlap between compound-related and disease-specific targets was used to construct a protein–protein interaction network using STRING, which was subsequently visualized in Cytoscape to identify hub genes. Molecular docking between stigmasterol and the top 3 hub genes was evaluated using the CB-Dock2 online server, and all preparations were performed in BIOVIA Discovery Studio. In vitro, Hep-G2 cells were treated with varying concentrations (0, 25, 50, 100, and 200 µg/mL) of *A. sinensis* extract. Cell viability, clonogenic potential, apoptosis induction, and migratory capacity were evaluated using the MTT assay, clonogenic assay, Annexin V/PI staining, and Transwell migration assay, respectively. Western blotting was used to assess the expression of key hub proteins in Hep-G2 cells.

**Results:**

In silico analysis identified an initial pool of 126 phytochemicals, which was refined to 4 key compounds yielding 139 unique targets. Intersection with HCC-related targets produced 123 common targets, generating a network comprising 119 nodes and 520 edges. STAT3, NFKB1, and TLR4 were identified as pivotal hub genes. Gene Ontology and Kyoto Encyclopedia of Genes and Genomes pathway analyses of the hub genes demonstrated significant enrichment in HCC-related pathways and biological processes. Molecular docking indicated a strong binding affinity of stigmasterol to STAT3, NFKB1, and TLR4, with binding energies of -7.8, -6.9, and -6.9 kcal/mol, respectively. In vitro assays showed a dose-dependent reduction in Hep-G2 cell viability and colony formation, significant induction of apoptosis, and marked inhibition of migration. Western blotting confirmed significant downregulation of STAT3, NFKB1, and TLR4 expression at higher extract concentrations.

**Conclusions:**

This study concluded that *A. sinensis* herbal extract was predicted to exert potent antiproliferative effects against HCC through the possible modulation of key signaling pathways and the targeting of hub genes, supporting its potential for further therapeutic development.

## 1. Background

Hepatocellular carcinoma (HCC) is a highly fatal and increasingly common primary liver cancer, representing a substantial proportion of global cancer-related mortality. Each year, 800,000 new cases of HCC are diagnosed, and more than 700,000 deaths are reported worldwide (1). Despite advances in medical therapies and early detection methods, the prognosis for HCC remains unfavorable, mainly because of underlying liver pathology, high recurrence rates after treatment, and limited therapeutic options for advanced-stage tumors (2). Conventional risk factors for HCC include chronic hepatitis infections, alcohol-induced liver disease, and, increasingly, nonalcoholic fatty liver disease, which is often associated with metabolic syndrome and obesity (3). In recent decades, the prevalence of HCC has increased, particularly in regions affected by these underlying conditions, underscoring the urgent need for novel approaches to drug development and tailored treatment strategies.

In parallel with the global health burden posed by HCC, traditional medicines remain an important source of potential therapeutic agents. *Angelica sinensis*, also known as Dong Quai or female ginseng, is a prominent medicinal plant that has been extensively used in traditional Chinese medicine for more than 2000 years (4). The dried root of *A. sinensis* is well regarded for its “blood-nourishing” properties and has traditionally been used to regulate menstruation, relieve discomfort, and enhance female reproductive health. Modern pharmacological research has expanded understanding of this plant by revealing a diverse phytochemical composition that supports its multiple medicinal properties. *Angelica sinensis* contains a wide range of bioactive constituents, including volatile oils, coumarins, ferulic acid, ligustilide, Z-ligustilide, polysaccharides, flavonoids, and organic acids (5). Ligustilide is considered the primary constituent responsible for several pharmacological actions, including anti-inflammatory, antioxidant, and neuroprotective properties (6). Butylidenephthalide (PubChem CID: 5352899) is a phthalide compound from *A. sinensis* with significant biological effects, including anti-inflammatory, anticancer, and cardiovascular protective effects. Other components, such as ferulic acid, enhance its capacity to regulate free radicals and promote vascular health (7).

Traditional herbal medicines pose challenges to contemporary drug development because of their intrinsic complexity. Unlike single-compound medicines, herbal extracts contain multiple constituents that can exert synergistic actions across diverse molecular pathways. Modern computational approaches, such as molecular docking and network pharmacology, are transforming this field (8, 9). Network pharmacology enables systematic examination of interactions among multiple active substances and their respective targets, thereby clarifying the comprehensive mechanisms of herbal formulations. By developing extensive drug-target-pathway networks, researchers can identify essential nodes that may serve as viable targets for therapeutic intervention in complex conditions (8). Molecular docking complements these network-based methodologies by modeling atomic-level interfaces between bioactive phytocompounds and target proteins (9). This approach provides critical insights into the binding affinity and stability of ligand-receptor complexes, which are essential for corroborating the therapeutic potential anticipated by network pharmacology investigations.

The increasing incidence of HCC and the complexity of traditional herbal therapies require renewed research approaches. The comprehensive phytochemistry of *A. sinensis*, characterized by diverse and stable bioactive components, underscores its potential to mitigate inflammatory and oxidative processes that contribute to liver carcinogenesis. Meanwhile, advanced approaches such as network pharmacology and molecular docking are transforming the drug development process through the comprehensive investigation of multitarget compounds. This integrated approach not only honors the legacy of traditional medicine but also represents a forward-looking strategy for combating modern diseases, ultimately bridging ancient wisdom and contemporary biomedical science.

## 2. Objectives

Hepatocellular carcinoma is a highly aggressive liver malignancy associated with a poor prognosis, limited effective therapeutic options, and an increasing global burden, underscoring the need to identify safer and more effective anticancer agents that target key molecular pathways. *Angelica sinensis* contains several bioactive compounds with reported pharmacological activities; however, its molecular mechanisms and therapeutic potential against HCC remain insufficiently explored. Therefore, this study used multiple computational approaches and in vitro evaluations to investigate the effects of *A. sinensis* on HCC.

## 3. Methods

### 3.1. Phytochemical Identification and Screening

Phytochemical compounds were initially identified using the Traditional Chinese Medicine Systems Pharmacology database (https://www.tcmsp-e.com/) (10). The Traditional Chinese Medicine Systems Pharmacology database is an integrative online platform that provides comprehensive data on herbal compounds and their pharmacokinetic properties by combining curated experimental results with predictive modeling. In this study, compounds were screened using oral bioavailability (OB) ≥ 30% and drug-likeness (DL) ≥ 0.18 to ensure that only compounds with favorable pharmacokinetic profiles were selected.

### 3.2. Target Identification

The SuperPred Target Prediction tool (prediction.charite.de/subpages/target_prediction.php) was used to screen biological targets of compounds that met the drug-likeness and bioavailability criteria. SuperPred is an online tool that uses chemical similarity searches and machine-learning algorithms to predict potential protein targets based on compound structure (28). Canonical SMILES structures of the compounds were downloaded from the PubChem database and submitted to SuperPred for target prediction. In parallel, HCC-related target genes were obtained from the GeneCards database (https://www.genecards.org/), an inclusive gene-centric resource (11). Intersecting targets between the predicted compound targets and HCC-associated targets were visualized using the jvenn online platform (http://jvenn.toulouse.inra.fr/), which generates Venn diagrams to efficiently display overlapping datasets (29).

### 3.3. Protein-Protein Interaction Network Generation

Intersecting targets identified from the overlap analysis were evaluated for protein-protein interactions using the STRING database (https://string-db.org/), which compiles known and predicted protein interactions and assigns confidence scores to these associations (12). The protein-protein interaction network was constructed for *Homo sapiens* with a confidence score of 0.400, and disconnected nodes were removed to streamline the network. The refined network was then imported into Cytoscape software (version 3.10.3; https://cytoscape.org/), where the cytoHubba plugin was used to identify hub genes based on topological features.

### 3.4. Ontology and Pathway Analysis

Gene Ontology analysis of intersecting genes was performed using the SRplot online platform (http://www.bioinformatics.com.cn/srplot), which integrates statistical analysis with interactive visualization tools to identify enriched biological processes, cellular components, and molecular functions (13). The platform includes a subtool, GO, pathway Enrichment Analysis, which was used to perform Gene Ontology and Kyoto Encyclopedia of Genes and Genomes enrichment analyses. The top 10 Gene Ontology terms and significant Kyoto Encyclopedia of Genes and Genomes pathways were identified, and a Sankey plot was generated to illustrate the relationships between enriched pathways and corresponding genes, thereby providing insights into functional interplay within the dataset.

### 3.5. Docking Analysis

Binding interactions of selected bioactive phytocompounds with target proteins were assessed by molecular docking against STAT3 (PDB ID: 6NJS), NFKB1 (PDB ID: 6QHD), and TLR4 (PDB ID: 8TQD). These structures were downloaded from the RCSB Protein Data Bank (https://www.rcsb.org/), a repository that provides high-resolution structural data for computational studies. The structures were prepared and energy-minimized using BIOVIA Discovery Studio (https://discover.3ds.com/discovery-studio), which provides tools for protein and ligand preparation, including optimization of molecular geometry and energy state to support accurate docking. Chain A of STAT3, NFKB1, and TLR4 was selected for docking simulations because it contained well-resolved and complete active/binding-site residues, which are critical for reliable ligand interaction analysis. Docking simulations were performed using the CB-Dock2 online platform (http://clab.labshare.cn/cb-dock2/), which automatically detects potential binding cavities on the protein surface and performs blind docking (14). Docking results were analyzed by evaluating cavity volume, binding energy estimates, and docking poses to identify the most promising ligand-protein complexes.

### 3.6. Molecular Dynamics Simulation

Of the 3 complexes, only the STAT3-stigmasterol complex was selected for molecular dynamics simulation because it had a better docking score than the other 2 complexes. This approach is consistent with standard practice, in which the top-ranked complex is prioritized for detailed molecular dynamics evaluation. The docked STAT3-stigmasterol complex was subjected to a 100 ns all-atom molecular dynamics simulation using GROMACS 2020. The protein was parameterized using the CHARMM36 force field, whereas ligand topology and parameters for stigmasterol were generated using the SwissParam server, which is compatible with CHARMM-based simulations. The system was solvated in a cubic box using the TIP3P water model, maintaining a minimum distance of 1.0 nm between the solute and box edges. Appropriate counterions were added to neutralize the system, followed by the addition of 0.15 M NaCl to mimic physiological conditions. Energy minimization was performed using the steepest descent algorithm until the maximum force was reduced below 1000 kJ mol^-1^ nm^-1^. Equilibration was performed in 2 phases: an NVT ensemble for 100 ps at 300 K using the V-rescale thermostat, followed by an NPT ensemble for 100 ps at 1 bar using the Parrinello-Rahman barostat. Long-range electrostatic interactions were treated using the Particle Mesh Ewald method, with a cutoff of 1.0 nm for both electrostatic and van der Waals interactions. The production molecular dynamics simulation was conducted for 100 ns with a 2 fs integration time step, applying periodic boundary conditions in all directions. Bond lengths were constrained using the LINCS algorithm, and trajectory frames were saved every 10 ps for subsequent analysis. Postsimulation analyses included root mean square deviation and root mean square fluctuation of Cα atoms (gmx rms/gmx rmsf), secondary-structure evolution (gmx do_dssp), protein-ligand interaction fractions (Maestro interface), ligand torsion profiles, radius of gyration, solvent-accessible surface area, polar surface area, and molecular surface area using built-in GROMACS and Schrödinger tools.

### 3.7. Chemicals and Reagents

*Angelica sinensis* herbal extract was obtained from Herbal Extract Co., Ltd. (123 Herbal Way, Beijing, China). The MTT reagent (3-[4,5-dimethylthiazol-2-yl]-2,5-diphenyltetrazolium bromide) was purchased from Sigma-Aldrich (St. Louis, MO, USA). Dimethyl sulfoxide, Dulbecco’s modified Eagle medium, fetal bovine serum, and antibiotics were obtained from Thermo Fisher Scientific (Waltham, MA, USA). The Annexin V-FITC and propidium iodide staining kit was sourced from BD Biosciences (Franklin Lakes, NJ, USA). Primary antibodies against STAT3, NFKB1, and TLR4 and their corresponding horseradish peroxidase-conjugated secondary antibodies were purchased from Cell Signaling Technology (Danvers, MA, USA). Transwell chambers (8 μm pore size) for the migration assay were procured from Corning Incorporated (Corning, NY, USA). Additional reagents for Western blotting, including SDS-PAGE gels, PVDF membranes, and chemiluminescent substrates, were purchased from Bio-Rad Laboratories (CA, USA).

### 3.8. Cell Culture

Human Hep-G2 cells were obtained from ATCC (Manassas, VA, USA) and cultured in Dulbecco’s modified Eagle medium supplemented with 10% fetal bovine serum, 100 U/mL penicillin, and 100 µg/mL streptomycin. Cells were maintained in a humidified incubator at 37°C with 5% CO_2_ and grown to 80% confluence to ensure optimal growth conditions and reproducibility in downstream assays.

### 3.9. Phytochemical Profile Using LC-MS

The dried A. sinensis extract was redissolved in methanol or 70% methanol at 1 - 5 mg/mL, vortexed, ultrasonicated for 30 - 60 minutes, and filtered through a 0.22 µm membrane before injection. UHPLC-Q-Orbitrap-MS or Q-TOF-MS analysis was performed on instruments such as a Thermo Vanquish UPLC with Q Exactive or an equivalent system, using a C18 reversed-phase column (eg, 2.1 × 100 mm, 1.7 - 2.6 µm particle size) at 30 - 35°C. Mobile phases consisted of 0.1% formic acid in water (A) and acetonitrile (B) at a 0.2 - 0.4 mL/min flow rate. A typical gradient elution was as follows: 0 - 5 minutes, 95% - 60% A; 5 - 15/20 minutes, 60% - 30/5% A; followed by washing at 5% A and re-equilibration to initial conditions, with a total run time of 20 - 35 minutes. The injection volume was 1 - 5 µL. ESI-MS was operated in positive and/or negative mode with a spray voltage of 3.0 - 3.5 kV (+)/-2.5 to -4.5 kV (-), a capillary temperature of 320 - 350°C, sheath/auxiliary gas of 35 - 60 arb units, full-scan m/z 100 - 1500, and data-dependent MS^2^ (collision energy, 10 - 40 eV, stepped). Compounds were identified based on accurate mass (< 5 ppm error), MS^2^ fragmentation, retention-time comparison, and reference to databases (mzCloud and HMDB) and the literature for signature constituents.

### 3.10. MTT Analysis

For the MTT assay, cells were plated in 96-well plates and allowed to adhere overnight before treatment with *A. sinensis* extract at concentrations of 0, 25, 50, 100, and 200 µg/mL. After a 24-hour treatment period, MTT reagent (0.5 mg/mL) was added to each well and incubated for 4 hours to enable formazan crystal formation. The crystals were then dissolved in dimethyl sulfoxide, and optical density was measured at 570 nm using a BioTek microplate reader (BioTek Instruments, Inc., Winooski, VT, USA) to quantify cell viability and metabolic activity.

### 3.11. Clonogenic Analysis

For the clonogenic assay, cells were seeded at low density in 6-well plates, allowed to attach overnight, and then exposed to extract concentrations of 0, 25, 100, and 200 µg/mL. Cells were incubated for 10 days to allow colony formation, after which they were fixed with methanol and stained with crystal violet. Colonies were visualized and counted using a Nikon Eclipse TS2 inverted microscope (Nikon Corporation, Shin Nihonbashi, Tokyo, Japan), providing high-resolution imaging for assessing long-term cell survival and proliferative capacity.

### 3.12. Annexin V/PI Staining Analysis

For apoptosis analysis, cells treated with 0, 25, 100, and 200 µg/mL of *A. sinensis* extract were collected and stained using an Annexin V-FITC/PI kit according to standard protocols. Stained cell populations were analyzed by flow cytometry on a BD FACSCanto II system (BD Biosciences, Franklin Lakes, NJ, USA). This analysis enabled differentiation of viable, early apoptotic, and late apoptotic or necrotic cells, providing detailed insight into the extract’s cytotoxic effects.

### 3.13. Transwell Assay

Cell migration was assessed using Transwell chambers (8 μm pore size). Cells treated with extract concentrations of 0, 25, 100, and 200 µg/mL were seeded in the upper chamber in serum-free medium. The lower chamber was filled with medium containing 10% fetal bovine serum as a chemoattractant. After 24 hours of incubation, cells that migrated through the membrane were fixed, stained with crystal violet, and quantified under a Nikon Eclipse TS2 inverted microscope (Nikon Corporation, Tokyo, Japan). This method provided a robust evaluation of the effect of the extract on cellular motility.

### 3.14. Western Blotting

For Western blot analysis of STAT3, NFKB1, and TLR4 expression, cells treated with 0, 25, 100, and 200 µg/mL of the extract were lysed in RIPA buffer supplemented with protease inhibitors. Protein concentrations were determined using the Bradford assay, and equal amounts were separated by 10% SDS-PAGE and transferred to PVDF membranes. After blocking with 5% nonfat milk, membranes were incubated with the specific primary antibodies and then with horseradish peroxidase-conjugated secondary antibodies. Protein bands were detected using a chemiluminescent substrate (Bio-Rad Laboratories, CA, USA). This approach provided quantitative and qualitative assessments of key signaling proteins involved in the cellular response to *A. sinensis* extract.

## 4. Results

### 4.1. Screening of Phytochemicals, Targets, and the Compound-Target Network

**Figure 1.**
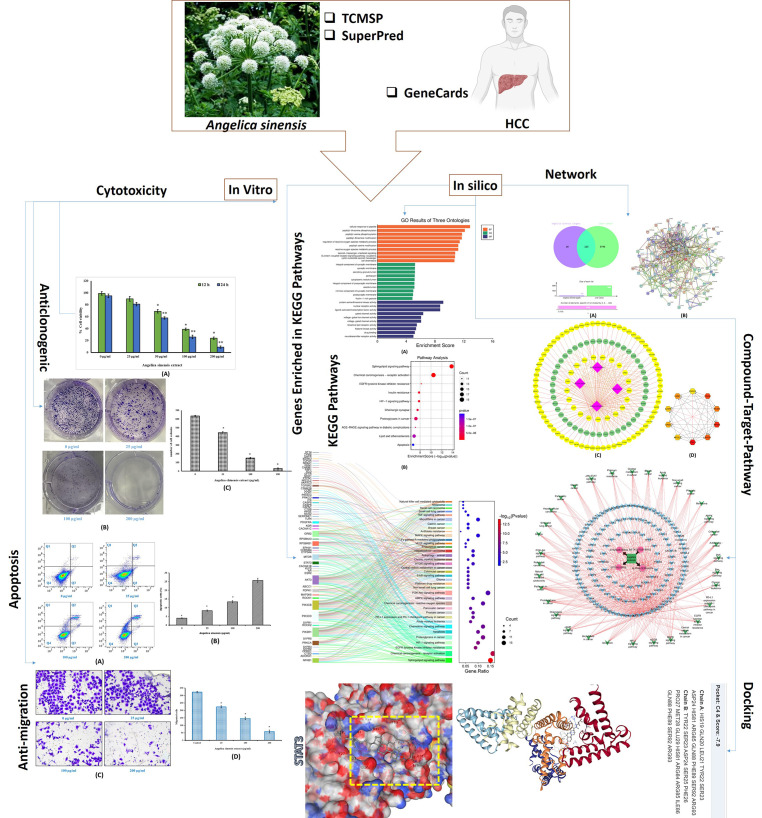
Comprehensive flow diagram showing the study outline and modus operandi adopted for the current research work.

The overall study design, methodology, and main findings are presented as a flow diagram in Figure 1.

Comprehensive phytochemical analysis using the Traditional Chinese Medicine Systems Pharmacology database initially identified 126 compounds from *A. sinensis*, which were subsequently refined to 4 key compounds—stigmasterol, sitosterol, alpha-cephalin, and sitogluside—based on stringent drug-likeness and oral bioavailability criteria (Table 1). Subsequent target prediction using SuperPred yielded 139 unique targets associated with these 4 compounds (Table 1 in the Supplementary File).

**Table 1.** Phytochemical Screening of *Angelica sinensis* From the TCMSP Database, Filtered to 4 Key Compounds (Stigmasterol, Sitosterol, Alpha-Cephalin, and Sitogluside) With Detailed Molecular Properties

Molecule Name	PubChem ID	Mol ID	MW	Hdon	AlogP	Hacc	Caco-2	OB (%)	DL	BBB	FASA-	HL
**Beta-sitosterol**	222284	MOL000358	414.79	1	8.08	1	1.32	36.91	0.75	0.99	0.23	5.36
**L-beta,gamma-dimyristoyl-alpha-cephalin**	114944	MOL008247	635.97	3	9.49	9	-0.43	20.69	0.47	-1.8	0.21	-
**Sitogluside**	5742590	MOL000357	576.95	4	6.34	6	-0.14	20.63	0.62	-0.93	0.23	-
**Stigmasterol**	5280794	MOL000449	412.77	1	7.64	1	1.44	43.83	0.76	1	0.22	5.57

### 4.2. Protein-Protein Interaction Network Construction and Analysis

**Figure 2.**
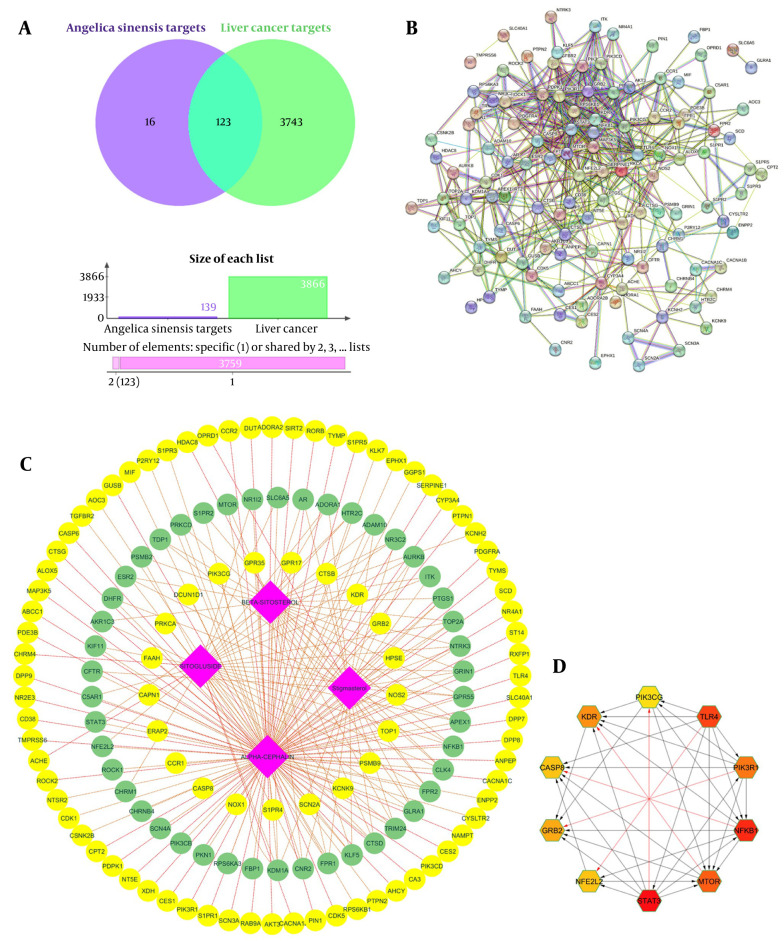
(A) Venn diagram illustrating the intersection between 139 compound-related targets and 3866 HCC-associated targets, resulting in 123 common targets. This figure visually represents the rigorous filtering and integration process that underpins subsequent network analyses. (B) Protein-protein interaction networks generated via STRING and (C) visualized in Cytoscape. These panels display a network of 119 nodes interconnected by 520 edges, reflecting the complex interactions among common targets and setting the stage for hub gene identification. (D) Degree centrality analysis of the protein-protein interaction network, highlighting the top 10 hub genes, including STAT3, NFKB1, and TLR4. This figure emphasizes the pivotal roles of these nodes within the network, suggesting their critical involvement in the pharmacological mechanisms of the extract.

In parallel, an extensive set of 19,097 HCC-related targets was compiled and refined to 3866 candidates by applying a GeneCards Inferred Functionality Score filter (Table 2 in the Supplementary File). Intersecting these targets with the 139 compound-related targets yielded 123 common targets (Figure 2A). These targets were entered into the STRING database to generate a protein-protein interaction network, which was then analyzed in Cytoscape and comprised 119 nodes interconnected by 520 edges (Figure 2B and C). Degree centrality analysis identified the top 10 hub genes—STAT3, NFKB1, TLR4, MTOR, PIK3R1, KDR, GRB2, NFE2L2, CASP8, and PIK3CG—highlighting their potentially critical roles in the pharmacological mechanisms of the extract (Figure 2D).

### 4.3. Functional Annotations

**Figure 3.**
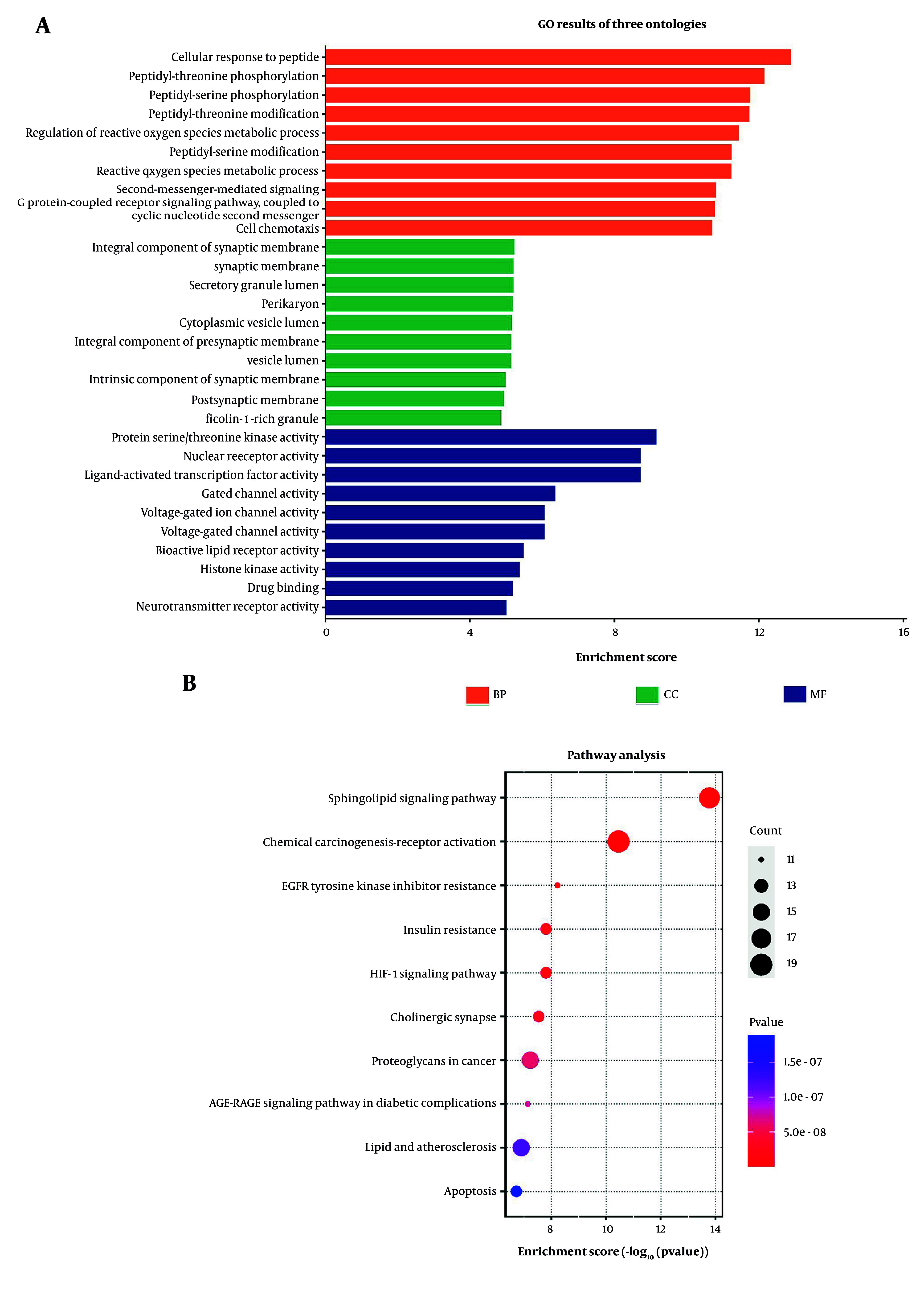
(A) Gene Ontology enrichment analysis depicting significantly enriched biological processes (eg, peptidyl phosphorylation and cellular response to peptide), cellular components, and molecular functions among the 123 common targets. This figure provides insight into the functional roles of these genes. (B) Kyoto Encyclopedia of Genes and Genomes pathway enrichment analysis showing key signaling pathways, such as sphingolipid signaling, EGFR tyrosine kinase inhibitor resistance, and apoptosis, that are significantly associated with the common targets. This highlights potential molecular mechanisms by which *A. sinensis* exerts its effects.

The common gene set was further subjected to Gene Ontology and Kyoto Encyclopedia of Genes and Genomes analyses via the SRplot platform. Enriched biological processes included cellular response to peptide; peptidyl-threonine and peptidyl-serine phosphorylation/modification; regulation of reactive oxygen species metabolic process; second messenger-mediated signaling; G-protein-coupled receptor signaling coupled to cyclic-nucleotide second messenger; and cell chemotaxis (Figure 3A). In the cellular component category, the targets were primarily associated with integral and intrinsic components of synaptic membranes, secretory granule lumen, perikaryon, and cytoplasmic vesicle lumen, among others (Figure 3A). Molecular function analysis highlighted activities such as PI3K activity; ligand-activated transcription factor and nuclear receptor activity; gated and voltage-gated ion channel activity; bioactive lipid receptor activity; and histone kinase activity (Figure 3A). Kyoto Encyclopedia of Genes and Genomes pathway analysis showed significant enrichment in sphingolipid signaling, chemical carcinogenesis via receptor activation, EGFR inhibitor resistance, insulin resistance, HIF-1 signaling, cholinergic synapse, proteoglycans in cancer, lipid and atherosclerosis pathways, and apoptosis (Figure 3B).

**Figure 4.**
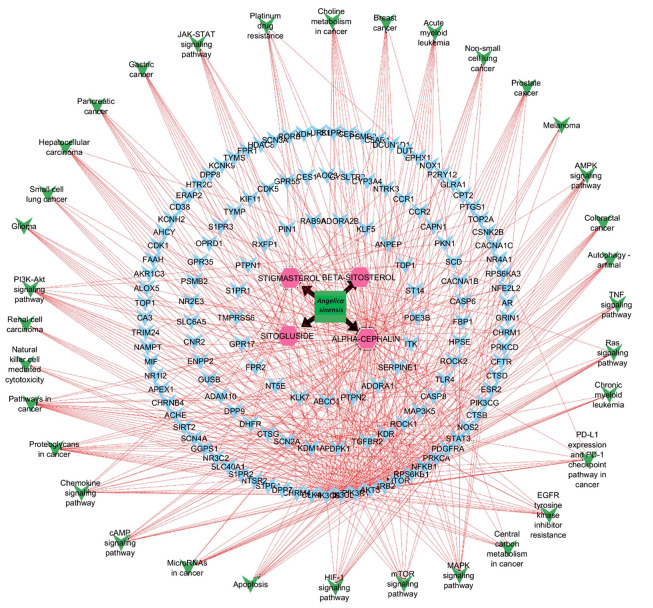
Compound-target-pathway network that integrates phytochemicals, common targets, and enriched Kyoto Encyclopedia of Genes and Genomes pathways. This comprehensive network illustrates the multifaceted mechanisms underlying the therapeutic potential of *A. sinensis*.

**Figure 5.**
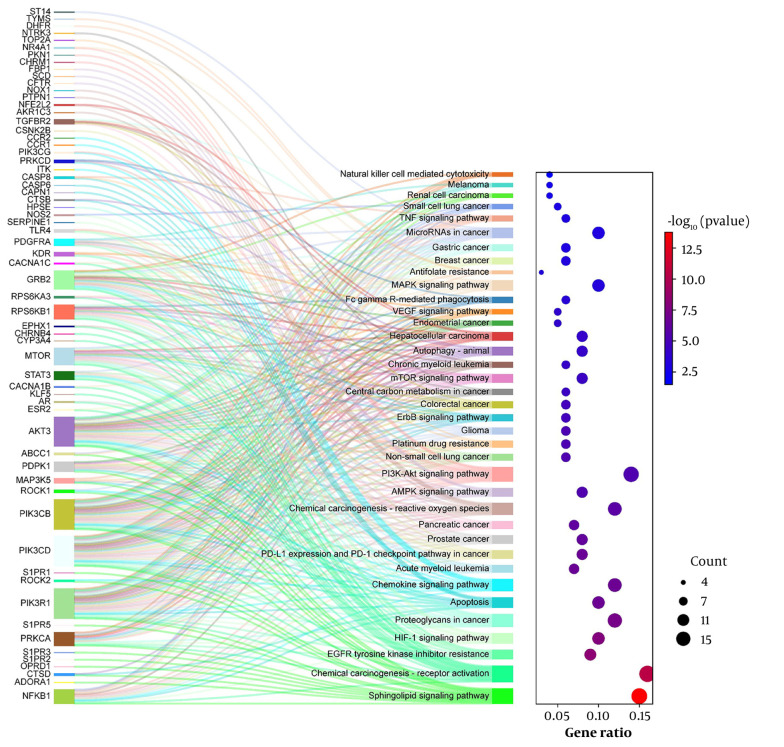
Sankey plot visualizing the relationships between enriched Kyoto Encyclopedia of Genes and Genomes pathways and their corresponding genes based on gene count and P value. This figure intuitively demonstrates the interconnectivity and relative impact of various pathways.

Collectively, these findings delineate the multifaceted molecular mechanisms underlying the therapeutic potential of *A. sinensis*, as illustrated by the compound-target-pathway network in Figure 4. The enriched genes in Kyoto Encyclopedia of Genes and Genomes pathways are presented as a Sankey plot based on gene count and P value (Figure 5).

### 4.4. Docking Validation for Hub Genes

**Figure 6.**
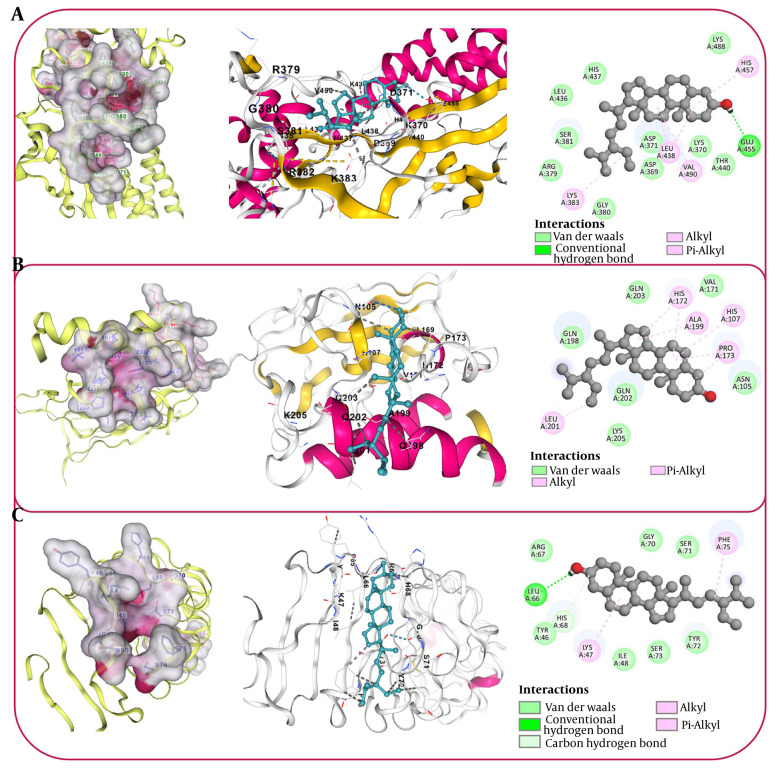
Molecular docking simulations illustrating significant binding interactions between stigmasterol and the top 3 hub proteins: (A) STAT3, (B) NFKB1, and (C) TLR4. The results support the hypothesis that stigmasterol may serve as a lead compound for targeting key signaling pathways in HCC.

Molecular docking studies further supported these findings, with stigmasterol demonstrating substantial binding interactions with the top 3 ranked target proteins, including STAT3, NFKB1, and TLR4, with binding energies of -7.8, -6.9, and -6.9 kcal/mol, respectively (Figure 6). Detailed docking simulations indicated that stigmasterol engages in strong interactions within the active sites of these proteins, exhibiting favorable binding affinities consistent with its prominent role in the compound-target network. This binding profile supports the hypothesis that stigmasterol acts as a lead compound for further drug development targeting key signaling pathways implicated in HCC.

### 4.5. Molecular Dynamics Simulation-Based Validation of Molecular Docking

**Figure 7.**
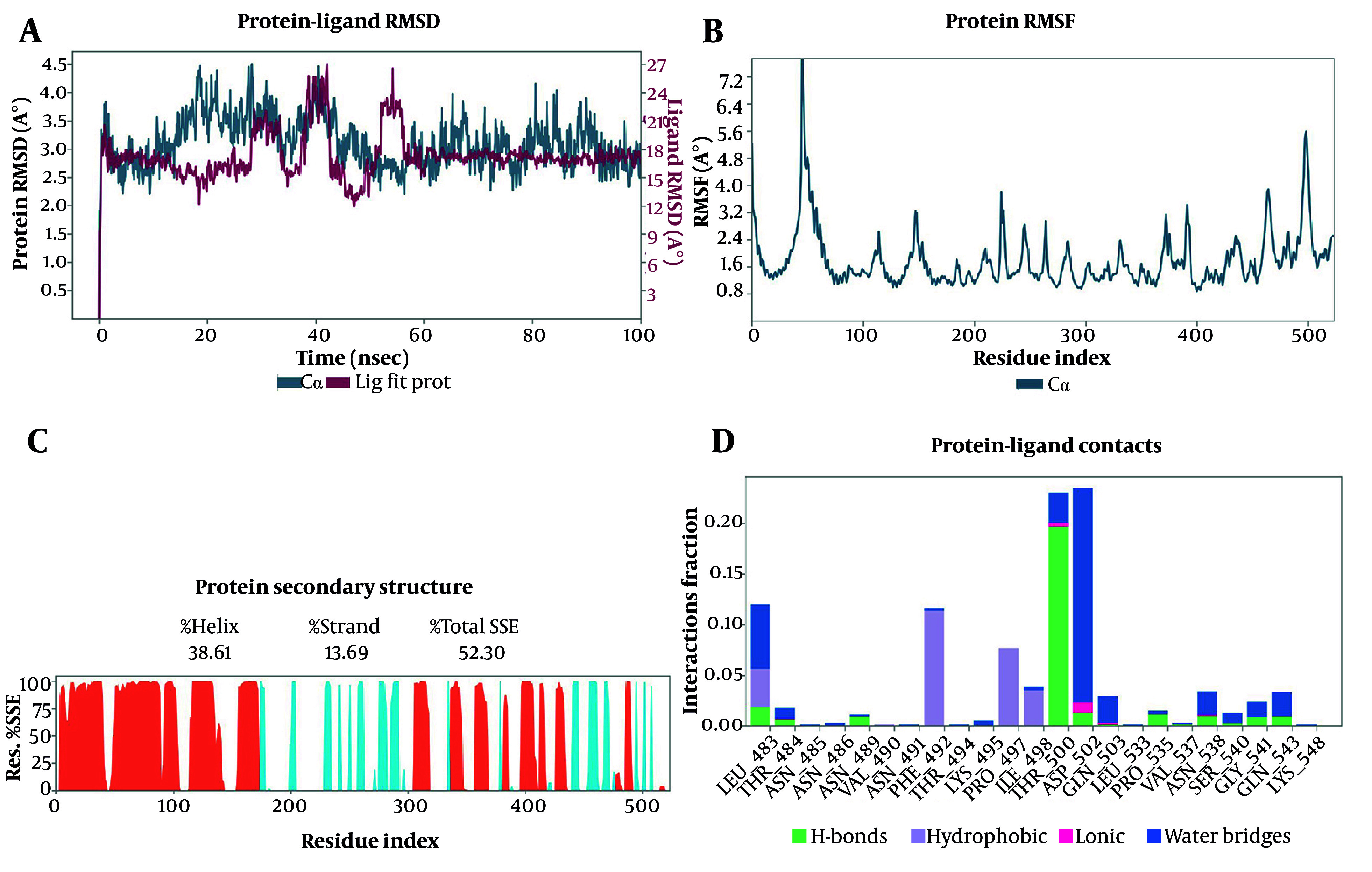
Molecular dynamics stability and interaction analysis of the STAT3-stigmasterol complex. (A) Time evolution of Cα root mean square deviation and ligand-fit root mean square deviation over 100 ns, demonstrating system equilibration and stable ligand binding after approximately 20 ns. (B) Residue-wise root mean square fluctuation profile showing minimal fluctuations in binding-site residues, with higher flexibility restricted to distal loop regions. (C) Secondary structural element analysis indicating consistent α-helix and β-strand content throughout the simulation. (D) Protein-ligand interaction timeline highlighting dominant contacts, including water bridges, hydrophobic interactions, and hydrogen bonds with key residues.

The 100 ns molecular dynamics trajectory of the STAT3-stigmasterol complex showed excellent stability, with protein Cα root mean square deviation plateauing at 2.5 - 3.0 Å and ligand-fit root mean square deviation remaining < 3.5 Å after 20 ns (Figure 7A), confirming the absence of dissociation or major conformational drift. Root mean square fluctuation analysis (Figure 7B) revealed low flexibility (< 2 Å) in binding-site residues and only transient peaks (7 Å) in distant loop regions (residues approximately 50 and 500), while secondary-structure content remained consistent at 38.61% α-helix, 13.69% β-strand, and 52.30% total secondary structural element throughout the simulation (Figure 7C). Persistent protein-ligand contacts (Figure 7D) were dominated by water bridges with ASP502 and GLN502, hydrophobic interactions with ILE498/LEU483, and occasional hydrogen bonds, all showing > 20% occupancy.

**Figure 8.**
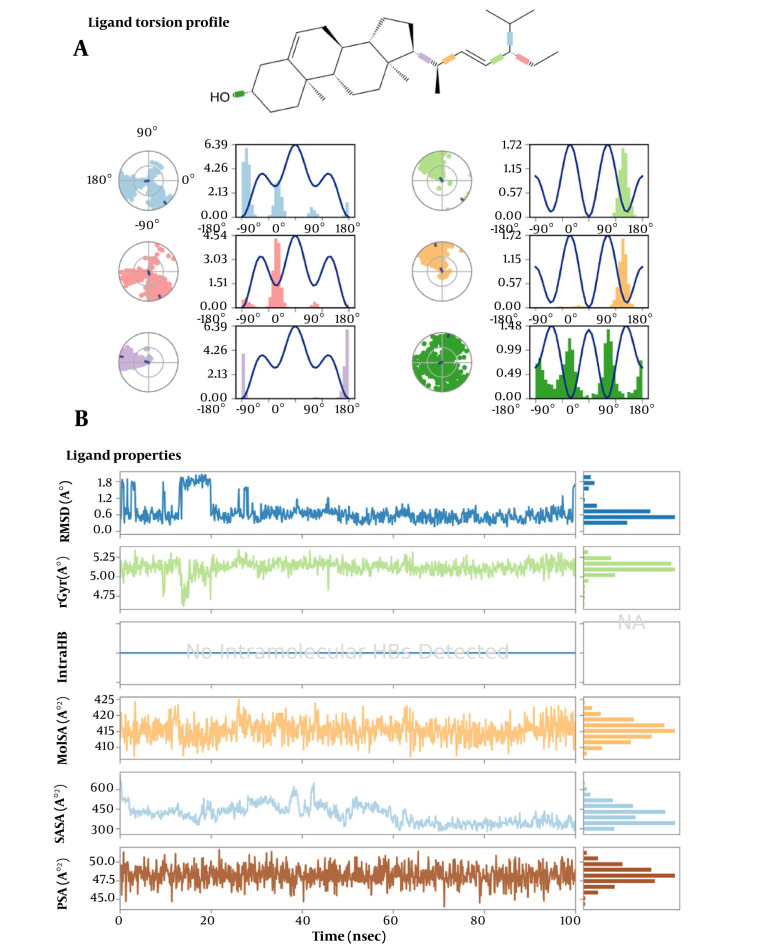
Conformational and physicochemical stability of stigmasterol during molecular dynamics simulation. (A) Ligand torsion angle analysis depicting stable dihedral behavior with minor conformational adjustments. (B) Ligand property evaluation, including root mean square deviation, radius of gyration, solvent-accessible surface area, and polar surface area, confirming a compact and equilibrated ligand conformation within the STAT3 binding pocket.

Ligand torsion profiles (Figure 8A) indicated stable dihedral angles with minor side-chain rotations, and ligand-property plots (Figure 8B) demonstrated low ligand root mean square deviation (0.6 - 1.2 Å), a constant radius of gyration, the absence of intramolecular hydrogen bonds, and equilibrated solvent-accessible surface area and polar surface area values. Collectively, these findings indicate that stigmasterol maintains a tightly bound, conformationally stable pose within the STAT3 pocket throughout the 100 ns simulation.

### 4.6. Validation of Phytochemical Presence Using LC-MS

**Figure 9.**
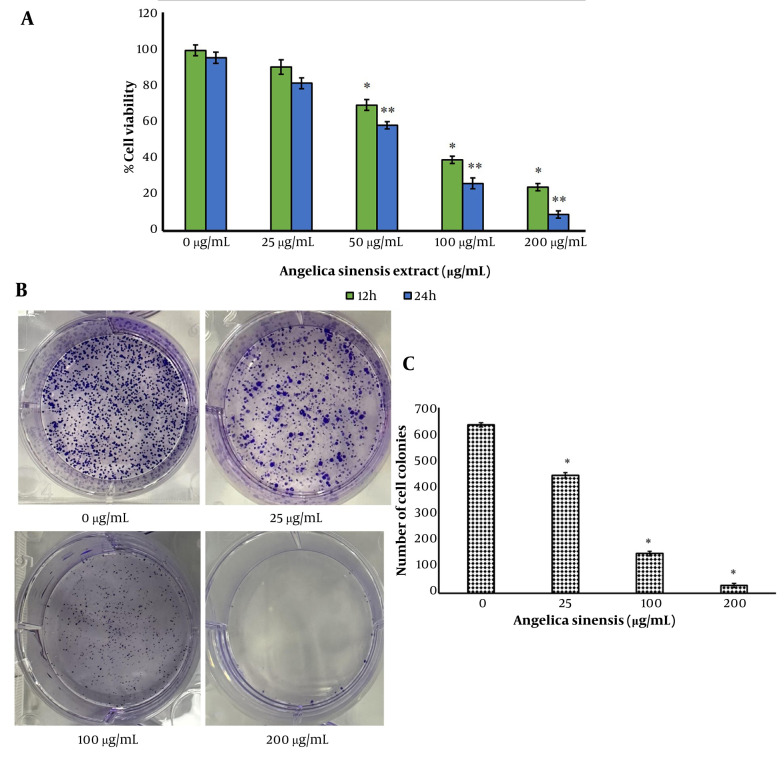
(A) Total ion chromatogram showing the separation and retention of key compounds, including sitoglucoside, α-cephalin, stigmasterol, and β-sitosterol, with stigmasterol eluting before β-sitosterol. The chromatogram also reveals multiple minor peaks corresponding to polar constituents, confirming the phytochemical diversity and analytical reliability of the extract. (B) MTT assay results showing a dose-dependent reduction in Hep-G2 cell viability, as reflected by decreasing viability with increasing concentrations of *A. sinensis* extract. (C) Representative culture plates from the clonogenic assay demonstrating dose-dependent inhibition of colony formation in Hep-G2 cells after treatment with the extract. (D) Quantitative analysis of colony formation, confirming significant reductions in colony numbers at higher extract concentrations, thereby indicating its antiproliferative effects.

LC-MS profiling of *A. sinensis* extract over a 35-minute run revealed a well-resolved and chemically diverse chromatogram, confirming the presence of 4 key compounds—sitoglucoside (β-sitosterol glucoside), α-cephalin, stigmasterol, and β-sitosterol—previously predicted through network-based analysis. Sitoglucoside was detected at 14.54 minutes, followed by α-cephalin at 19.28 minutes, whereas stigmasterol (20.22 minutes) and β-sitosterol (21.12 minutes) appeared as dominant high-intensity peaks. The earlier elution of stigmasterol relative to β-sitosterol is consistent with its higher degree of unsaturation and reduced retention on C18 columns (Figure 9A). Their identities were further supported by characteristic fragment ions. Additional minor peaks corresponded to polar constituents such as phthalides, phenolics, and organic acids, underscoring the phytochemical complexity of the extract. Collectively, the sharp peak resolution, stable baseline, and reproducible ion signatures validate analytical robustness and provide experimental confirmation of network-predicted bioactive sterols within a diverse phytochemical matrix.

### 4.7. Cytotoxic and Cell Colony-Inhibiting Effects of *Angelica sinensis*

In Hep-G2 cells, treatment with *A. sinensis* extract induced a pronounced, dose-dependent decrease in cell viability, as determined by the MTT assay (Figure 9B). Cells exposed to increasing extract concentrations (0 - 200 µg/mL) showed a significant reduction in metabolic activity, with the highest concentrations (100 and 200 µg/mL) yielding markedly lower absorbance values at 570 nm than those of the control group (P < 0.05). This reduction in viability underscores the cytotoxic potential of the extract in Hep-G2 cells.

The clonogenic assay further supported these findings, revealing dose-dependent inhibition of colony formation. Hep-G2 cells treated with 0 - 200 µg/mL of the extract formed progressively fewer colonies over a 14-day period (Figure 9C). Notably, significantly fewer colonies were observed at 100 and 200 µg/mL (Figure 9D), indicating that the extract not only reduces short-term cell viability but also impairs the long-term proliferative capacity of these cells (P < 0.05).

### 4.8. Apoptotic Cytotoxicity Induced by *Angelica sinensis*

**Figure 10.**
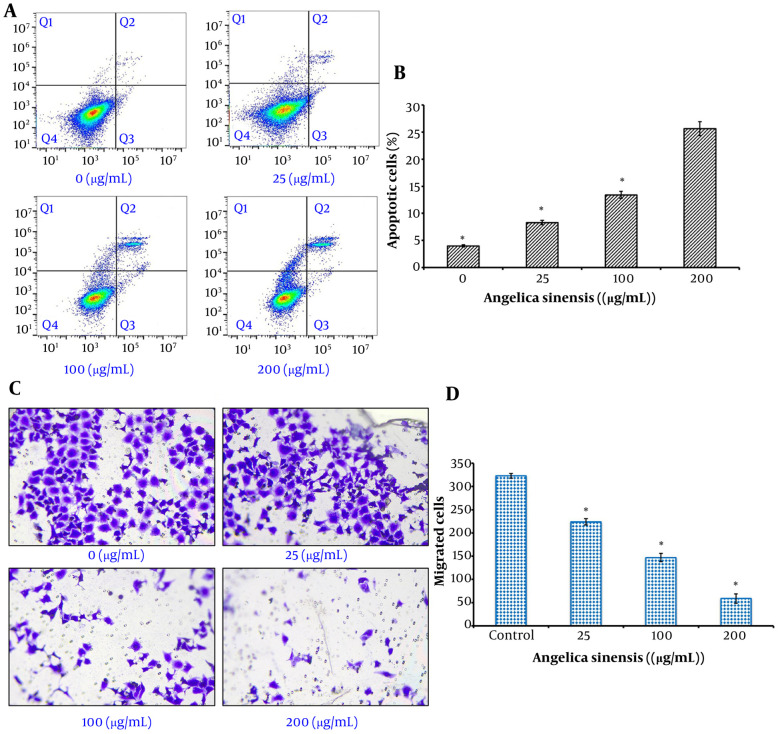
(A) Flow cytometry dot plots from Annexin V/PI staining revealing a dose-dependent increase in apoptotic cell populations in Hep-G2 cells treated with *A. sinensis* extract. (B) Bar graph quantifying apoptotic cell percentages, highlighting significant increases in both early and late apoptosis with higher extract doses. (C) Representative images from the Transwell migration assay showing diminished cell migration in Hep-G2 cells treated with escalating concentrations of the extract. (D) Quantitative analysis of the migration assay data, illustrating a significant dose-dependent decrease in the number of migrating cells, underscoring the antimetastatic potential of the extract.

Flow cytometry demonstrated a clear, dose-dependent induction of cell death (Figure 10A). With increasing extract concentration, both early and late apoptotic cell populations increased concomitantly (P < 0.05) (Figure 10B). Hep-G2 cells exposed to 200 µg/mL of the extract exhibited the highest percentage of apoptotic cells, suggesting that the cytotoxic effect of *A. sinensis* is mediated, at least in part, through activation of apoptotic pathways.

### 4.9. Anti-Migration Effects of *Angelica sinensis*

The Transwell migration assay provided further insight into the antimetastatic potential of the extract. Hep-G2 cell migration was significantly (P < 0.05) impaired in a dose-dependent manner (Figure 10C). When treated with 0, 25, 100, and 200 µg/mL of the extract, the number of cells migrating through the 8 μm pores progressively declined (Figure 10D). This reduction in cell motility indicates that *A. sinensis* extract effectively inhibits the migratory capability of Hep-G2 cells, which is critical for its potential role in mitigating metastasis.

### 4.10. Hub Gene Expression

**Figure 11.**
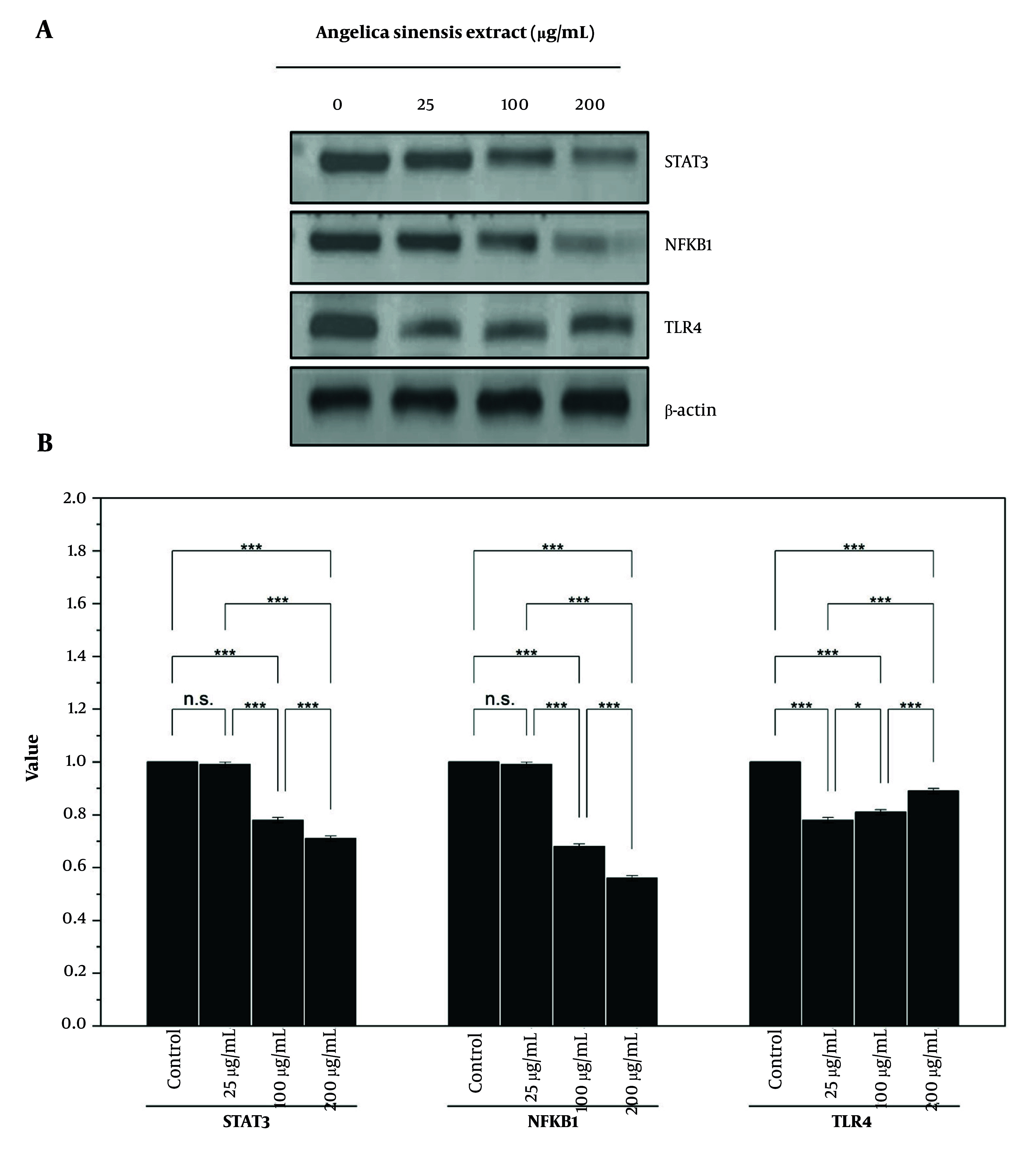
(A) Western blots displaying the expression levels of STAT3, NFKB1, and TLR4 in Hep-G2 cells treated with various concentrations of *A. sinensis* extract. (B) Densitometric analysis of Western blot bands showing dose-dependent downregulation of STAT3, NFKB1, and TLR4, corroborating the modulatory effects of the extract on key signaling proteins.

Western blot analysis of key signaling proteins, STAT3, NFKB1, and TLR4, showed that their expression levels were modulated in a dose-dependent manner after treatment with *A. sinensis* extract (Figure 11A). Specifically, densitometric analysis demonstrated significant downregulation of STAT3, NFKB1, and TLR4 protein expression at higher extract concentrations (100 and 200 µg/mL) relative to untreated controls (Figure 11B). This suppression of pivotal regulatory proteins suggests that the extract interferes with major signaling pathways involved in cell survival, proliferation, and inflammatory responses in Hep-G2 cells.

## 5. Discussion

Network construction integrated compound targets derived from Traditional Chinese Medicine Systems Pharmacology and SuperPred with HCC-related targets filtered from GeneCards. The resulting interaction network, based on degree centrality analysis, identified pivotal hub genes such as STAT3, NFKB1, and TLR4, underscoring their importance in key signaling pathways. This approach provided a strong foundation for therapeutic target identification and enabled a systematic understanding of the molecular processes underlying HCC.

STAT3, NFKB1, and TLR4 are pivotal mediators in cancer biology, orchestrating complex signaling networks that contribute to tumor initiation, progression, and resistance to therapy. STAT3 (signal transducer and activator of transcription 3) is a transcription factor that, when activated via phosphorylation through the JAK/STAT pathway, translocates to the nucleus to regulate proliferation, survival, and angiogenesis (15, 16). Aberrant STAT3 signaling has been observed in various cancers, including breast, lung, and colorectal cancers, in which constitutive activation promotes oncogenic transformation and tumor maintenance (17). The ability of STAT3 to upregulate antiapoptotic and pro-proliferative genes makes it a central node in oncogenic signaling networks and a promising target for anticancer therapies (18). Accordingly, the literature indicates that STAT3 is an important target in cancer therapy, and our network analysis identified it as the top interacting gene in the protein-protein interaction network. Thus, our network analysis directly corroborates prior studies.

NFKB1 (nuclear factor kappa-B subunit 1) is another key transcription factor that forms part of the NF-κB complex, which is essential for regulating immune and inflammatory responses (19, 20). In cancer, persistent NF-κB activation has been linked to enhanced cell survival, resistance to apoptosis, and chronic inflammation, all of which contribute to tumor development and progression (21). In malignancies such as lymphoma, pancreatic cancer, and prostate cancer, NF-κB signaling fosters an environment conducive to tumor growth by promoting the expression of genes that inhibit programmed cell death and stimulate proliferation (22). Crosstalk with other signaling pathways further amplifies its oncogenic potential. We identified NFKB1 as a key hub gene in the protein-protein interaction network, aligning our results with the existing literature and the pathways it modulates, including apoptosis.

TLR4 (Toll-like receptor 4) is traditionally known for its role in innate immunity through recognition of molecular patterns linked to pathogens; however, emerging evidence indicates that TLR4 is also intricately involved in cancer (23). TLR4 signaling can initiate inflammatory cascades that inadvertently support tumorigenesis through activation of downstream effectors, including NF-κB and STAT3 (24). In several cancer types, including ovarian, gastric, and hepatocellular carcinoma, TLR4 engagement has been associated with enhanced carcinoma survival and metastasis. The ability of this receptor to modulate the tumor microenvironment by recruiting inflammatory cells further underscores its significance in promoting a protumorigenic milieu. TLR4 has been shown to be critical in modulating cellular processes that enable evasion of cell death and support tumorigenesis. Our bioinformatics analysis identified TLR4 as a central hub gene in the protein-protein interaction network, predicting it as a high-value target in HCC.

Collectively, the interplay among STAT3, NFKB1, and TLR4 contributes to a robust network of oncogenic signaling pathways. Their activation not only sustains cancer cell growth and survival but also facilitates evasion of apoptosis and promotes angiogenesis and metastasis. Targeting these molecules has therefore become an attractive strategy in the development of novel cancer therapeutics aimed at disrupting the signaling cascades underlying tumor progression and resistance mechanisms.

The coordinated regulation of cytotoxicity, apoptosis, and metastasis by STAT3, NFKB1, and TLR4 is central to the malignant phenotype observed in many cancers (25). Activation of STAT3 often induces transcription of genes that promote survival and proliferation while inhibiting apoptotic processes. Consequently, its dysregulation can reduce cytotoxicity in response to cellular stress or therapeutic agents. Similarly, NFKB1 drives the expression of antiapoptotic factors, thereby conferring resistance to cell death. Its persistent activation creates an environment in which cancer cells can withstand cytotoxic insults and evade apoptosis.

TLR4, by initiating innate immune responses, can inadvertently enhance metastatic potential by modulating the tumor microenvironment (26). It activates downstream signaling cascades, such as those involving STAT3 and NF-κB, leading to the secretion of pro-inflammatory cytokines that facilitate tumor cell migration and invasion. In addition to promoting metastasis, this inflammatory environment also increases chemoresistance (27). Moreover, interplay among these pathways orchestrates epithelial-mesenchymal transition, a key metastatic process. In sum, the combined aberrant activation of STAT3, NFKB1, and TLR4 disrupts normal cellular homeostasis, reduces cytotoxic efficacy, inhibits apoptosis, and fosters metastatic behavior in cancer cells.

In the context of our study, STAT3, NFKB1, and TLR4 emerged as hub genes, underscoring their central role in orchestrating key oncogenic pathways. Our in silico analyses revealed that these genes not only serve as critical nodes within the protein-protein interaction network but also exhibit robust interconnectivity with other molecular targets implicated in HCC. The prominence of STAT3 in the network aligns with its established role in promoting proliferation and survival in liver cancer cells, whereas the involvement of NFKB1 reflects its contribution to an inflammatory microenvironment that supports tumor progression. TLR4, by mediating innate immune responses, further highlights the crosstalk between inflammation and carcinogenesis in HCC.

Our findings demonstrated that treatment with *A. sinensis* extract resulted in a significant, dose-dependent reduction in the viability of Hep-G2 cells. This cytotoxic effect was accompanied by downregulation of the identified hub genes, STAT3, NFKB1, and TLR4. Suppression of these key regulatory genes, which are critically involved in oncogenic signaling pathways governing proliferation and survival, suggests that their knockdown may contribute to the observed reduction in cell viability. *Angelica sinensis* extract significantly enhanced apoptotic cell death in Hep-G2 cells. The induction of apoptosis was associated with downregulation of the hub genes STAT3, NFKB1, and TLR4, which are known to regulate inflammatory signaling and apoptosis resistance in HCC. Knockdown of these genes may disrupt pro-survival signaling networks, thereby facilitating activation of apoptotic pathways. In addition, *A. sinensis* extract markedly inhibited the migratory capacity of Hep-G2 cells. This anti-migratory effect was correlated with downregulation of STAT3, NFKB1, and TLR4, genes that play pivotal roles in tumor invasion, inflammation-mediated signaling, and metastatic progression. Knockdown of these hub genes may impair downstream signaling cascades that regulate cytoskeletal dynamics, epithelial-mesenchymal transition, and cell motility. These results demonstrate the therapeutic potential of *A. sinensis* extract in HCC, with STAT3, NFKB1, and TLR4 emerging as key molecular targets whose suppression may contribute to reduced proliferation, enhanced apoptosis, and diminished migratory behavior of cancer cells. These results warrant further mechanistic and in vivo studies to validate the potential of these targets for the development of novel therapeutic strategies against HCC.

### 5.1. Limitations

This study has notable limitations that temper interpretation of the findings. First, experiments were conducted exclusively in the Hep-G2 HCC cell line without inclusion of additional HCC cell lines or normal hepatocytes as controls. This design restricts generalizability and precludes assessment of potential selectivity or off-target effects in nonmalignant liver cells. Second, the in vitro downregulation of STAT3, NFKB1, and TLR4 by *A. sinensis* extract remains correlative; the study did not include functional validation, such as siRNA/shRNA knockdown, overexpression rescue experiments, or specific pathway inhibitors, to establish direct causality between the hub genes and the observed antiproliferative, pro-apoptotic, and anti-migratory effects. These constraints underscore the need for broader cell-line panels, normal-cell controls, and mechanistic follow-up studies to strengthen the proposed therapeutic rationale.

### 5.2. Conclusions

In summary, this integrative study elucidated the antiproliferative mechanisms of *A. sinensis* extract against HCC. Network analysis identified 123 common targets, with STAT3, NFKB1, and TLR4 as hub genes, whereas Gene Ontology and Kyoto Encyclopedia of Genes and Genomes analyses revealed involvement in peptide phosphorylation, inflammatory signaling, sphingolipid signaling, and apoptosis. Molecular docking validated strong binding of stigmasterol to key proteins. In vitro assays demonstrated dose-dependent cytotoxicity, reduced colony formation, increased apoptosis, and inhibited migration in Hep-G2 cells, with Western blotting confirming the downregulation of pivotal signaling proteins. Collectively, these results indicate the multifaceted antiproliferative potential of the herbal extract against the HCC cell line Hep-G2 and merit further investigation.

ijpr-25-1-169852-s001.zip

## Data Availability

The dataset presented in the study is available on request from the corresponding author during submission or after publication.
